# Seizure semiology and predictors of outcomes in Chinese patients with glutamic acid decarboxylase antibody-associated neurological syndrome

**DOI:** 10.1186/s12883-023-03182-x

**Published:** 2023-04-11

**Authors:** Nan Lin, Lin Bai, Qing Liu, Jianhua Chen, Haitao Ren, Hongzhi Guan, Qiang Lu

**Affiliations:** Department of Neurology, Peking Union Medical College Hospital, NO.1 Shuaifuyuan Hutong of Dongcheng District, Beijing, 100730 China

**Keywords:** GAD antibody associated neurological syndrome, Seizure, Outcome, Treatment, EEG

## Abstract

**Background:**

In the current study, seizure semiology and potential predictive factors of seizure outcomes in glutamic acid decarboxylase antibody (GAD Ab)-associated neurological syndrome were investigated.

**Methods:**

In this study, 32 Chinese patients with GAD Ab-associated neurological syndrome who presented with seizures at Peking Union Medical College Hospital from January 2017 to October 2022 were reviewed; 30 had a follow-up duration of more than 1 year.

**Results:**

Among the 32 patients, 10 presented with epilepsy alone. Concomitant neurological syndromes were observed in 22 patients, including limbic encephalitis (*n* = 20), stiff-person syndrome (SPS, *n* = 1), and cerebellar ataxia (*n* = 1). Bilateral tonic–clonic seizures were observed in 21 patients (65.6%). Focal seizures occurred in 27 patients (84.4%); 17 had focal motor seizures and 18 focal non-motor seizures. Among 30 patients with long-term follow-up, 11 (36.7%) were seizure-free. Acute/subacute onset (*p* = 0.049) and comorbidity of limbic encephalitis with epilepsy (*p* = 0.023) led to better seizure outcomes. Patients with persistent epilepsy were more likely to have focal seizure (*p* = 0.003) and higher frequency of seizure (*p* = 0.001). Furthermore, these patients tended to have longer intervals from onset to immunomodulatory treatments. Early immunotherapy (within 6 months from onset) was administered in 81.8% of seizure-free patients but only in 42.1% of patients with persistent seizures. However, steroid and immunosuppressant duration did not differ in the two groups. Repeated serum GAD Ab tests during the follow-up showed no association with seizure outcomes.

**Conclusions:**

The seizure manifestations are diverse and variable. Approximately one third of patients achieved seizure remission during long-term follow-up. The type and frequency of seizures may influence the seizure outcomes. Early immunotherapy, especially within 6 months, may lead to better seizure outcomes.

**Supplementary Information:**

The online version contains supplementary material available at 10.1186/s12883-023-03182-x.

## Background

Glutamic acid decarboxylase (GAD) is an enzyme for gamma-aminobutyric acid (GABA) synthesis. GAD antibodies (Abs) were first described in 1988 by Solimena et al. [1]. Subsequently, patients with various neurological disorders and type 1 diabetes mellitus (T1DM) presented with GAD Abs, which comprise the GAD Ab-spectrum disorders [2]. The main associated neurological syndromes were stiff-person syndrome (SPS), cerebellar ataxia, epilepsy, and limbic encephalitis [3].

Approximately 11.5%–14.9% GAD Ab-associated patients have epilepsy [3, 4], especially temporal lobe epilepsy [5]. Epilepsy can present alone or coexist with cerebellar ataxia and SPS. These patients are more likely to have therapy-refractory focal epilepsy of long duration [6]. Errichiello et al*.* found GAD Abs were present in 2.6% of patients with epilepsy [7], most frequently in patients with chronic pharmacoresistant epilepsy that involves the temporal lobes [8].

A single-center retrospective study of seizures was performed in Chinese patients with GAD Ab-associated neurological syndromes in China to identify semiology and predictive factors associated with seizure outcome. The results could provide useful information regarding the treatment and clinical monitoring in GAD Ab-associated epilepsy patients.

## Methods

### Patients

A retrospective study was used to analyze the clinical characteristics and predictors of seizure outcomes. The clinical database of Peking Union Medical College Hospital (PUMCH) from January 2017 to October 2022 was searched. Patients with positive serum/cerebrospinal fluid (CSF) anti-GAD Abs were identified. The inclusion criteria were seizures, with or without other neurological symptoms, and exclusion of other central neural system diseases that may influence seizure outcome. Finally, 32 patients were included and 2 patients were lost to follow-up. The follow-up period for the 30 patients ranged from 1–5 years.

Seizures were evaluated by at least one experienced epilepsy specialist according to the new classification of seizures by the International League Against Epilepsy (ILAE) 2017 [9]. Paroxysmal subjective feeling without epileptiform discharges in EEG recordings and no response to anti-seizure medication (ASM) or immunomodulatory therapies were not considered seizures.

Diagnosis of autoimmune limbic encephalitis was based on the following criteria established by Graus et al*.* [10]: (1) subacute onset (< 3 months) of memory deficits, seizures, or psychiatric symptoms suggesting involvement of the limbic system; (2) bilateral brain abnormalities on T2-weighted fluid-attenuated inversion recovery (FLAIR) MRI highly restricted to the medial temporal lobes or; (3) at least one of the following: CSF pleocytosis (white blood cell count of more than five cells per mm^3^), EEG epileptic or slow-wave activity involving the temporal lobes; (4) exclusion of other causes. In patients with positive CSF/serum GAD Ab, diagnosis can be made when two of the first three criteria have been met and other causes are excluded. Patients with chronic seizures and minor memory deficits were not diagnosed with limbic encephalitis regardless of the MRI findings. For patients who presented only recurrent seizures and no bilateral MRI temporal lobes lesions, if the disease courses were more than three months and the seizure frequency was not acute/subacute exacerbated in the first three months, then the isolated epilepsy was made in these patients.

The presence of anti-GAD65 Abs was evaluated using a fixed cell-based indirect immunofluorescence test with BIOCHIP (Euroimmun AG, Lüebeck, Germany). The cell-based analysis test steps were performed as follows: 30 μl of the plasma (dilution fold 1:10) or CSF was added to the thin slices of the biological substrates on the sample plate, incubated for 30 min at room temperature and flushed with phosphate buffer solution. Then they were incubated with 25 μl of sheep anti-IgG labeled with FITC for 30 min and rinsed with PBS. The results of tests were evaluated under a fluorescence microscope.

### Statistical analysis

Continuous non-normal distribution data were described as median with interquartile range (IQR). Mann–Whitney *U* test was performed for group comparisons. The frequency data between the two groups were compared using chi-square test or Fisher’s exact test. All tests were two-tailed and statistical significance was set at *p* = 0.05.

## Results

A total of 32 patients were included in the current study and 24 patients were female (Table 1). The median age at onset of seizures was 41 years (IQR 27–52 years) and 3 patients were under 18 years of age. Three patients were acute onset (< 2 weeks), 18 were subacute onset, and 11 was chronic onset. More than half of the patients developed memory impairment (*n* = 19). Other clinical symptoms included psychiatric disorders (*n* = 9), sleep disorders (*n* = 7), movement disorders (*n* = 5), fever (*n* = 4), consciousness disorders (*n* = 4), ataxia (*n* = 2), diplopia (*n* = 1), and speech disorders (*n* = 1). The initial symptom included seizures (*n* = 26), psychiatric symptoms (*n* = 2), consciousness disorders (*n* = 1), memory deficits (*n* = 1), ataxia (*n* = 1) and speech disorders (*n* = 1). The accompanied neurological syndromes included limbic encephalitis (*n* = 20), SPS (*n* = 1), and cerebellar ataxia (*n* = 1). Ten patients presented with epilepsy alone, 4 of whom had a history of drug-resistant epilepsy. One patient developed persistent tongue and jaw clonus after bilateral tonic–clonic seizures at onset; the oral clonus disappeared after administration of intravenous diazepam. Complications of other diseases occurred in 17 patients and included diabetes (*n* = 7; T1DM, *n* = 5), chronic thyroiditis (*n* = 6), hypothyroidism (*n* = 3), Graves disease (*n* = 2), and vitiligo (*n* = 2). Among the 7 patients with coexistent diabetes, 5 patients had positive CSF GAD Ab. Another two patients with positive GAD Ab by cell-based assay also had performed chemiluminescence GAD assays with the values > 2000 IU/ml. With epilepsy and other neurology symptoms, the diagnosis of GAD antibody associated neurological syndrome was made in these 7 patients.

**Table 1:** Clinical characteristics and treatments

	Total (*n *= 32)	Group 1 (*n* = 11)	Group 2 (*n* = 19)	P_1-2_
**Demographics**				
Age (seizure onset, median, IQR)	41 (27-52)	43 (31-52)	35 (25-53)	0.112
Gender (M:F)	8:24	3:8	5:14	1.000
**Disease onset**				
Acute/subacute (<3m)	21 (65.6%)	10 (90.9%)	10 (52.6%)	0.049
Chronic	11 (34.4%)	1 (9.1%)	9 (47.4%)	
**Seizure characteristics**				
Tonic-clonic seizures	21 (65.6%)	9 (81.8%)	11 (57.9%)	0.246
Focal seizures	27 (84.4%)	6 (54.5%)	19 (100%)	0.003
Seizure frequency	*n* = 26	*n* = 10	*n* = 14	0.001
Daily	10	1	9	
Weekly	3	0	3	
Monthly	9	6	2	
<1 time per month	4	3	0	
**Other symptoms**				
Fever	4 (12.5%)	0	3 (15.8%)	0.279
Memory impairment	19 (59.4%)	10 (90.9%)	9 (47.4%)	0.023
Psychosis	9 (28.1%)	3 (27.3%)	6 (31.6%)	1.000
Sleep disorders	7 (21.9%)	2 (18.2%)	4 (21.1%)	1.000
Movement disorder	5 (15.6%)	1 (9.1%)	4 (21.1%)	0.626
Consciousness disorder	4 (12.5%)	2 (18.2%)	1 (5.3%)	0.537
Speech dysfunction	1 (3.1%)	0	1 (5.3%)	1.000
Ataxia	2 (6.2%)	1 (9.1%)	1 (5.3%)	1.000
Arrhythmia	1 (3.1%)	0	1 (5.3%)	1.000
Diplopia	1 (3.1%)	1 (9.1%)	0	0.367
**Overall neurological syndromes**				
Limbic encephalitis	20 (62.5%)	10 (90.9%)	9 (47.4%)	0.023
Cerebellar ataxia	1 (3.1%)	0	1 (5.3%)	1.000
Stiff-person syndrome	1 (3.1%)	0	1 (5.3%)	1.000
Isolated epilepsy	10 (31.2%)	1 (9.1%)	9 (47.4%)	0.049
**Investigations**				
Baseline Serum GAD Ab	*n* = 29	*n* = 10	*n* = 17	1.000
1:32	2 (6.9%)	1 (10%)	1 (5.9%)	
1:100	20 (69.0%)	7 (70%)	12 (70.6%)	
1:320	7 (24.1%)	2 (20%)	4 (23.5%)	
CSF	*n* = 20	*n* = 8	*n* = 12	
Baseline CSF GAD Ab				
1:32	5 (25%)	1(12.5%)	4(33.3%)	0.405
1:100	7(35%)	3(37.5%)	4(33.3%)	
1:320	8(40%)	4(50%)	4(33.3%)	
WBC (/uL, median, IQR)	1 (0-4)	1 (0-2)	2 (0-10)	0.432
Increased protein	5 (25%)	2 (25%)	3 (25%)	1.000
EEG recordings	*n* = 25	*n* = 8	*n* = 16	
Normal	4 (16%)	2 (25%)	2 (12.5%)	0.578
Epileptiform discharge	18 (72%)	4 (50%)	13 (81.2%)	0.167
Temporal	18(72%)	4(50%)	13(81.2%)	0.167
Extra-temporal	2(8%)	0	2(12.5%)	0.536
Slow wave	11 (44%)	3 (37.5%)	7 (43.7%)	1.000
Temporal	6 (24%)	3 (37.5%)	3(18.7%)	0.362
Diffused slow wave	5 (20%)	0	4 (25%)	0.262
Brain MRI	*n* = 24	*n* = 9	*n* = 14	
Normal	7 (29.2%)	2 (22.2%)	4 (28.6%)	1.000
Medial temporal lobe	17 (70.8%)	7 (77.8%)	10 (71.4%)	1.000
Bilateral	10 (41.7%)	6 (66.7%)	3 (21.4%)	0.077
Other	3 (12.5%)	1 (11.1%)	2 (14.3%)	1.000
PET/CT	*n* = 8	*n* = 1	*n* = 7	
Abnormal	6 (75%)	0	6 (85.7%)	0.250

*GAD* Glutamic acid decarboxylase, *IQR *Interquartile range

At seizure presentation, 21 (65.6%) patients had bilateral tonic–clonic seizures; 16 showed focal onset and 5 showed only tonic–clonic seizures of unknown origin (Table 1). Focal seizure was present in 27 (84.4%) patients; 17 (63.0%) had impaired awareness and 17 (63.0%) had focal motor seizures. Eighteen (66.7%) patients had focal non-motor seizures manifesting with palpitation (*n* = 7), dyscognitive state (*n* = 6), sensory disorders (*n* = 6; head region, *n* = 4; hemi-lateral body, *n* = 3), goosebumps (*n* = 3), fear/sadness (*n* = 3), déjà vu (*n* = 2), visual hallucination (*n* = 2), nausea (*n* = 2), and olfactory hallucinations (*n* = 1). Musciogenic seizure was observed in a 37-year-old female patient. The seizure manifested with fear, sadness, nausea, goosebumps, and hand automatism. Excluding 6 patients who receiving more than one year ASM treatments, seizure frequencies were evaluated before both ASM and immunomodulatory therapies in the other 26 patients. Daily seizures (> 1 time per day) occurred in 10 (38.5%) patients, 3 (11.5%) showed weekly seizures (> 1 time per week), 9 (34.6%) had monthly seizures (> 1 time per month), and 4 (15.4%) patients had < 1 seizure per month.

EEG recordings were available in 25 patients (Table 1) and results were normal in 4 (16%) patients. Epileptiform discharges were observed in 18 (72%) cases and all occurred in temporal regions. Multifocal epileptiform discharges were observed in 2 patients, including occipital and parietal lobes. Clinical attacks were detected in 10 patients during EEG recording, with focal motor seizures in 4 patients and focal non-motor seizures in 8 patients. Seizures originating from the temporal lobe were observed in 9 patients and the other patient who presented with persistent tongue and jaw clonus had normal EEG results except muscle artifacts of unknown origin. Slowing waves were observed in 11 patients, including local regions (temporal lobe, *n* = 6) and diffuse slow wave (*n* = 5).

All patients had positive serum/CSF GAD Abs (Table 1). Serum GAD Ab titer results were obtained from 29 patients, and CSF GAD Ab results from 20 patients. Abnormal CSF results were found in 6 patients (elevated protein, range 0.47–0.53 g/L, *n* = 5; increased white cells, range 12–18 cells/μL, *n* = 3).

**Fig. 1:**
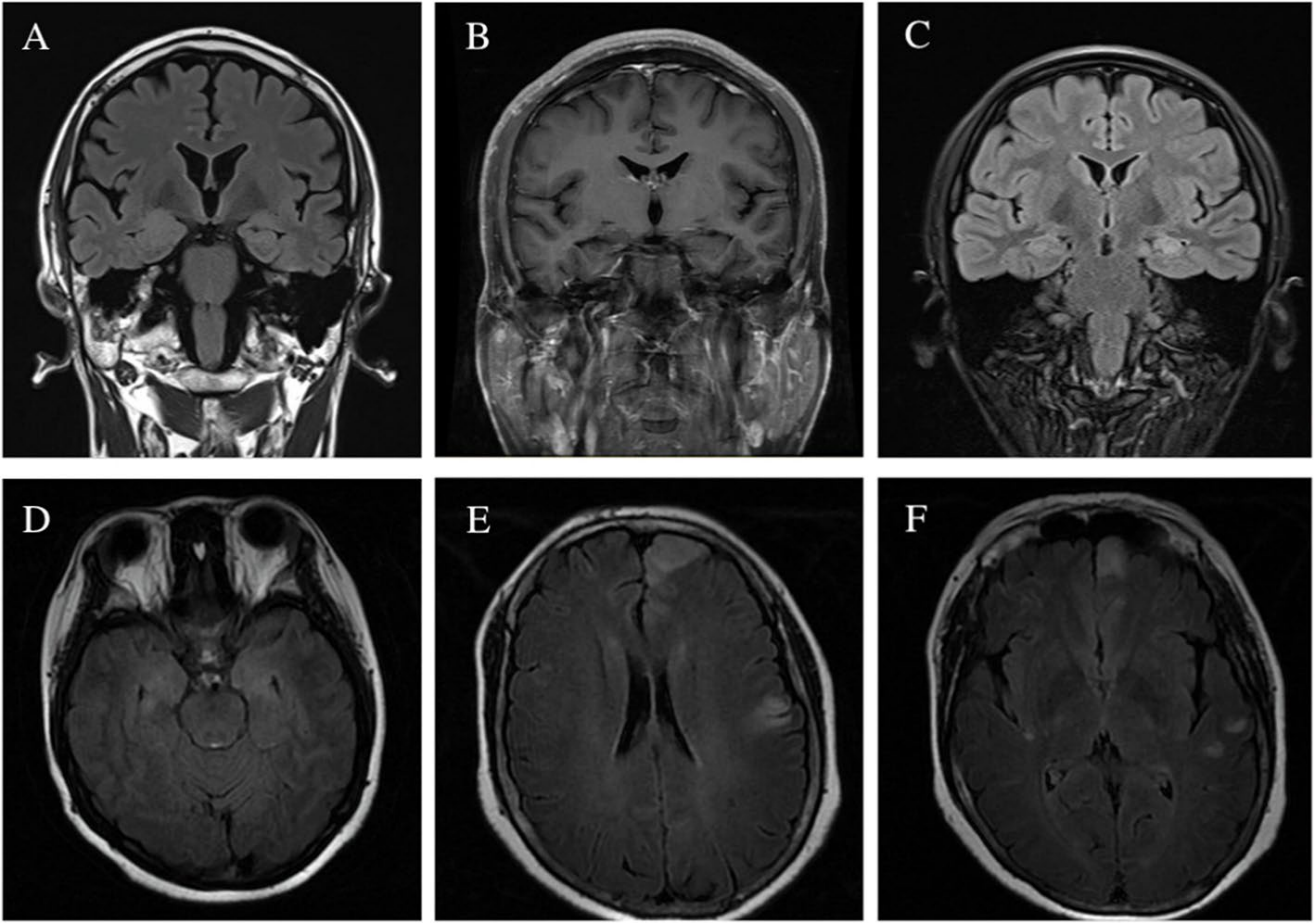
MRI images in patients with GAD antibody associated seizures. **A** Right hippocampal swelling and T2 hyperintensity in a 59-year-old male in 4 months from onset. **B** Right hippocampal atrophy in a 23-year-old male in 4 years from onset. **C** Left hippocampal T2 hyperintensity in a 19-year-old female in 2 years from onset. **D** T2 hyperintensity in bilateral mesial temporal lobes in a 43-year-old female in one month from onset. **E**–**F** Multiple cortical/subcortical T2 hyperintensities in frontal, parietal, temporal and insular lobes in a 54-year-old female in one month from onset

Brain MRI was available in 24 cases and 7 patients had normal results. All 17 (70.8%) abnormal MRI results had T2 hyperinternsities in mesial temporal lobe, bilaterally in 10 and unilaterally in 7. Seven of them showed hippocampal swelling (performed MRI within 6 months) and 3 had hippocampal atrophy (performed MRI more than 2 years). Brain MRI revealed multiple region lesions in 3 patients involving frontal lobe, cingulate gyrus, thalamus, insular and occipital lobe (Fig. 1). Eight patients underwent 18F-FDG PET/CT; 6 (75%) had abnormal metabolic pattern involving temporal lobes (*n* = 6), cortex of other lobes (*n* = 2), basal ganglia (*n* = 1), cerebellar (*n* = 1), and whole brain (*n* = 1).

ASM was used by 27 patients. The median number of ASM administered to each patient was 2 (IQR, 1–3). Levetiracetam was the most used ASM (*n* = 17), followed by oxcarbazepine/carbamazepine (*n* = 13), valproate (*n* = 8), lamotrigine (*n* = 3), topiramate (*n* = 2), lacosamide (*n* = 2), and perampanel (*n* = 2). All 32 patients received immunomodulatory therapies, including intravenous immunoglobulin (IVIG, a course of 2 g/kg, repeated if necessary according to clinical conditions) and steroids. Immunosuppressants were administered to 22 patients; 21 were administered mycophenolate mofetil (MMF) and 1 cyclophosphamide. The therapy changed from azathioprine to MMF in 1 patient due to poor treatment response. After a follow-up period > 1 year, 11 (36.7%) patients were seizure-free, 9 experienced > 50% reduction in seizures, including the patient with musicogenic reflex seizure, 10 had < 50% reduction in seizures, including 1 patient with SPS and 1 patient with cerebellar ataxia and the other 2 patients were lost to follow-up. One patient received anterior temporal lobotomy due to the poor immunomodulatory therapy response. After surgery, the frequency of seizure attack decreased from several times per day to 1–2 times per week. Repeated serum GAD Ab tests were performed in 25 patients; 11 showed decreased GAD Ab titer and 9 had unchanged or increased GAD Ab titer. The serum GAD Ab level was decreased but reincreased in another 5 patients, of whom 2 had relapse.

**Fig. 2:**
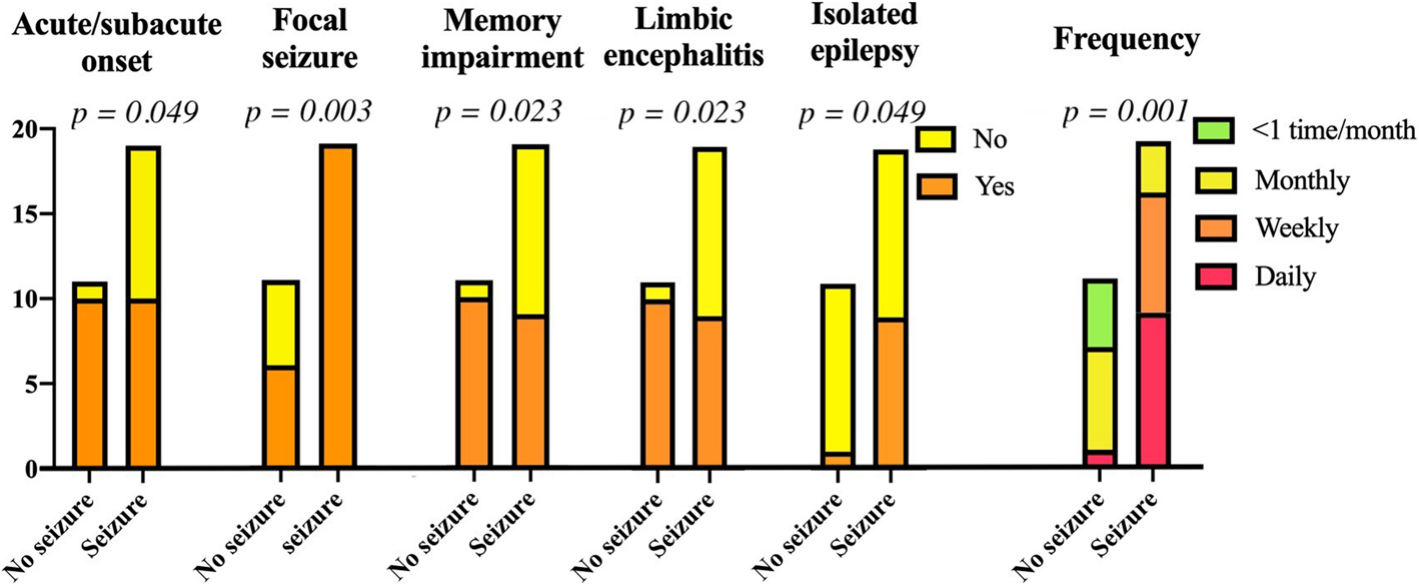
Factors influenced on the seizure outcomes. More patients in seizure group were chronic onset and had focal seizures and isolated epilepsy, while memory deficits and limbic encephalitis were more common in no seizure group. Patients with seizure-free had less frequent seizure attacks before immunotherapy

The 30 patients with follow-up records were divided into two groups: seizure-free group (group 1, *n* = 11) and seizure group (group 2, *n* = 19). As shown in Table 1, the two groups had no significant difference in terms of age or gender. Compared to group 1, more chronic onset and less acute/subacute onset occurred in group 2 (*p* = 0.049). More patients had memory impairment in group 1 than in group 2 (*p* = 0.023). Comorbidity of limbic encephalitis with epilepsy led to better seizure outcomes (*p* = 0.023). Patients with epilepsy alone had poor seizure control (*p* = 0.049). In addition, focal seizure was more common in group 2 compared with group 1 (Table 1, *p* = 0.003). Seizure attacks in group 2 were more frequent than in group 1 (*p* = 0.001). The factors which influenced the outcome were illustrated in Fig. 2. Group comparisons showed no significant differences in other clinical symptoms. CSF examinations and brain MRI results showed no significant differences between groups.

The intervals from onset to steroid and IVIG treatments tended to be longer in group 2 than in group 1 (Table 2). Early immunomodulatory therapy (starting within 6 months from disease onset) was administered to 9 (81.8%) patients in group 1 and 8 (42.1%) patients in group 2, indicating that starting earlier immune therapy tended to result in better seizure outcome; however, the difference was non-significant (*p* = 0.057). Steroid treatment duration did not differ in the two groups. Patients in group 2 tended to have longer immunosuppressant treatment duration, possibly due to the persistent seizure attack. Repeated serum GAD Ab testing during the follow-up showed no significant group difference. Four patients in seizure group had repeated CSF GAD Ab, which was increased in 2 patients and decreased in 2 patients.

**Table 2:** Treatments and outcome in anti-GAD encephalitis

	Group 1(*n* = 11)	Group 2(*n* = 19)	P
Early immunomodulatory therapy (<6m)	9 (81.8%)	8 (42.1%)	0.057
IVIG	8 (72.7%)	16 (84.2%)	0.641
Start time (/m) (median, IQR)	4.25 (1-5)	7.5 (2-45)	0.291
Steroid	10 (90.9%)	16 (84.2%)	1.000
Start time (/m) (median, IQR)	4.25 (1.5-6)	8 (2-48)	0.517
Steroid duration (/m) (median, IQR)	12 (4-18)	8 (6-14)	0.537
Immunosuppressants duration(/m) (median, IQR)	15 (10-24)	21 (9-24)	0.563
GAD Ab	*n* = 9	*n* = 16	
Titers from last follow-up (1-4y)			0.806
1:10	2 (22.2%)	0	
1:32	3 (33.3%)	8 (42.1%)	
1:100	2 (22.2%)	7 (36.8%)	
1:320	2 (22.2%)	1 (5.3%)	
Change from baseline			0.600
Decreased	4 (44.4%)	7 (43.7%)	
Unchanged/increased	2 (22.2%)	7 (43.7%)	
Decreased-->Increased	3 (33.3%)	2 (12.5%)	
ASM	9 (81.8%)	17 (89.5%)	0.358
Number of ASM (median, IQR)	1 (1-1)	2 (1-3)	0.040

*GAD* Glutamic acid decarboxylase, *IVIG* Intravenous Immunoglobulin, *IQR* Interquartile range, *ASM* Anti-seizure medicine

In summary, patients who had acute/subacute onset, limbic encephalitis and memory impairment showed better seizure outcomes. Isolated epilepsy, focal seizures and frequent seizure attacks indicated poor seizure controls. Early immunomodulatory therapy (< 6 m) may lead to better seizure controls.

## Discussion

This was a retrospective cohort study that included 32 Chinese patients with GAD Ab-associated seizures. In this study, 75% of patients were female, which was consistent with previous studies, and the median age was 41 years, older than the reported median age (26 years at onset of seizures) [8]. Approximately 70% of patients had concomitant clinical symptoms, most commonly with memory impairment, followed by psychosis and sleep disorders. More than half of the patients had limbic encephalitis. However, overlap syndrome with SPS and cerebellar ataxia was observed in only 6% of patients, a little higher than the reported percentage of 2.9% in limbic encephalitis/epilepsy [11].

Memory impairment and limbic encephalitis occurred more often in group 1 than in group 2. More patients presented with epilepsy alone in group 2 compared with group 1, possibly because memory deficits and limbic encephalitis may lead to early immunomodulatory treatment. In the present study, > 80% of patients in group 1 started immunomodulatory therapy within 6 months from onset and in group 2, < 50% of patients received early immunotherapy. IVIG and steroid treatment tended to be administered earlier in group 1 than in group 2. In previous studies, early immunotherapy was reportedly associated with better outcomes based on a series of 6 cases [8, 12]. For patients with poor response to ASM and immunotherapy, surgery after assessments using EEG, MRI and PET/CT could be a choice.

Nearly 30% patients had normal brain MRI. Abnormal MRI results showed no influence in seizure outcome. The most common imaging finding is mesial temporal lobe T2 hyperintensity (70.8%), while previous study reported hippocampal involvement only were seen in a minority of patients [13]. Multiple lobes cortical/subcortical T2 hyperintensity was observed in 12.5% patients, which was less than previous reported [13]. Furthermore, GAD Ab titers at the time of diagnosis did not predict seizure outcome. Repeated tests during follow-up revealed that serum GAD Ab titer did not correlate with seizure severity; similar results were previously reported [2, 14].

Focal seizures were observed in 84.4% of patients. The difference in focal seizure occurrence and frequency between the two groups indicates that frequent focal seizures may be associated with poor seizure control. The semiology manifestations were diverse. Musicogenic reflex seizure, a rare form of epilepsy induced by sounds, was observed in 1 patient. Musicogenic seizure and positive GAD Abs were previously reported in 4 cases [15–17]. Among these 5 patients, the median age at seizure onset was 37 years (30–56 years) and 4 were female. All 5 patients showed responses to ASM or immunomodulatory therapy but were not seizure-free.

In the present study, 1 patient presented with persistent oral clonus and bilateral tonic–clonic seizures. The clonus could be stopped by administration of diazepam. However, the EEG recording was normal. The origin of oral clonus was unknown. Cases of rhythmic palatal myoclonus and palatal jerks of undetermined origin associated with GAD Abs have been previously described [18, 19]. Jaw jerk was absent in both cases. For patients with newly developed the clonus of facial and oropharyngeal muscles, GAD Ab test should be considered.

Half of the patients had other diseases, which was consistent with a previous study [20]. The prevalence of diabetes in patients with GAD Ab-associated seizures was 22%, higher than the prevalence of 11.2% in the Chinese population [21]. GAD is widely expressed in the central nervous system and pancreatic b-cells. Anti-GAD Abs are found in T1DM patients and in first-degree relatives and are a marker for predicting the risk of diabetes [22]. Furthermore, approximately a third of the study cohort had thyroid disease, including chronic thyroiditis, hypothyroidism, or Graves disease, and 6.2% had vitiligo. For patients with GAD Abs, blood glucose monitoring should be regularly performed regardless of diabetes history. In addition, primary evaluations should include testing for thyroid function and thyroid-associated antibodies.

### Limitations

This is a retrospective cohort of patients with GAD Ab associated seizures. Prospective research should be set up in the future, which could provide more powerful and convincing evidence of the seizure outcome predictors. Secondly, the sample size of seizure-free group was small, which may affect the statistical analysis of group comparisons.

## Conclusions

The seizure manifestations in patients with GAD Ab-associated neurological syndromes are diverse and variable. Seizures can manifest with epilepsia partialis continua in addition to acute symptoms of limbic encephalitis and temporal epilepsy. After immunotherapy and ASM treatment, approximately one third of patients achieved seizure remission. Overlap with limbic encephalitis may be associated with better seizure control. Frequent focal seizures may be a factor for poor seizure control. Early immunotherapy, especially starting within 6 months after onset, may result in better seizure outcomes. However, the duration of steroid and immunosuppressant treatments was not associated with seizure control.

## Supplementary Information

**Additional file 1: Table S1. **Clinical characteristics and treatments. **Table** **S2. **Treatments and outcome in anti-GAD encephalitis.

## Data Availability

The datasets used and/or analysed during the current study are available from the corresponding author on reasonable request.

## Abbreviations

GAD: Glutamic acid decarboxylase
GABA: Gamma-aminobutyric acid
Abs: Antibodies
T1DM: Type 1 diabetes mellitus
SPS: Stiff-person syndrome
CSF: Cerebrospinal fluid
ASM: Anti-seizure medication
MMF: Mycophenolate mofetil
