# Supplementary material for: Seizure semiology and predictors of outcomes in Chinese patients with glutamic acid decarboxylase antibody-associated neurological syndrome
Source: BMC Neurol. 2023 Apr 11;23:149. doi: 10.1186/s12883-023-03182-x (PMC10088211; doi:10.1186/s12883-023-03182-x)
Supplement: Supplementary file 1 — Additional file 1: Table S1. Clinical characteristics and treatments. Table S2. Treatments and outcome in anti-GAD encephalitis. [file 12883_2023_3182_MOESM1_ESM.docx]

**Table 1.** Clinical characteristics and treatments.

|  | Total  (n=32) | Group 1  (n = 11) | Group 2  (n = 19) | P_1-2_ |
| --- | --- | --- | --- | --- |
| Age (seizure onset) | 37 (6-71) | 41 (19-71) | 29 (12-63) | 0.112 |
| Gender (M:F) | 8:24 | 3:8 | 5:14 | 1.000 |
| Tonic-clonic seizures | 21 (65.6%) | 9 (81.8%) | 11 (57.9%) | 0.246 |
| Focal seizures | 27 (84.4%) | 6 (54.5%) | 19 (100%) | 0.003 |
| Seizure frequency  Daily  Weekly  Monthly  <1 time per month | 11  7  9  5 | 1  0  6  3 | 9  7  3  0 | <0.001 |
| Fever | 4 (12.5%) | 0 | 3 (15.8%) | 0.279 |
| Memory impairment | 19 (59.4%) | 10 (90.9%) | 9 (47.4%) | 0.023 |
| Psychosis | 9 (28.1%) | 3 (27.3%) | 6 (31.6%) | 1.000 |
| Sleep disorders | 7 (21.9%) | 2 (18.2%) | 4 (21.1%) | 1.000 |
| Movement disorder | 5 (15.6%) | 1 (9.1%) | 4 (21.1%) | 0.626 |
| Consciousness disorder | 4 (12.5%) | 2 (18.2%) | 1 (5.3%) | 0.537 |
| Ataxia | 2 (6.2%) | 1 (9.1%) | 1 (5.3%) | 1.000 |
| Stiff-person syndrome | 1 (3.1%) | 0 | 1 (5.3%) | 1.000 |
| Speech dysfunction | 1 (3.1%) | 0 | 1 (5.3%) | 1.000 |
| Arrhythmia | 1 (3.1%) | 0 | 1 (5.3%) | 1.000 |
| Diplopia | 1 (3.1%) | 1 (9.1%) | 0 | 0.367 |
| Limbic encephalitis | 20 (62.5%) | 10 (90.9%) | 9 (47.4%) | 0.023 |
| Isolated epilepsy | 10 (31.2%) | 1 (9.1%) | 9 (47.4%) | 0.044 |
| Serum GAD Ab  1:32  1:100  1:320 | n=29  2 (6.9%)  20 (69.0%)  7 (24.1%) | n=10  1 (10%)  7 (70%)  2 (20%) | n=17  1 (5.9%)  12 (70.6%)  4 (23.5%) | 1.000 |
| CSF | n=20 | n=8 | n=12 |  |
| GAD Ab  1:32  1:100  1:320 | 5 (25%)  7(35%)  8(40%) | 1(12.5%)  3(37.5%)  4(50%) | 4(33.3%)  4(33.3%)  4(33.3%) | 0.405 |
| White cells (/uL) | 1 (0-18) | 1 (0-4) | 2 (0-18) | 0.432 |
| Increased protein | 5 (25%) | 2 (25%) | 3 (25%) | 1.000 |
| EEG recordings  Normal  Epileptiform discharge  Slow wave | n=25  4 (16.7%)  18 (72%)  11 (44%) | n=8  2 (25%)  4 (50%)  3 (37.5%) | n=16  2 (13.3%)  13 (81.2%)  7 (43.7%) | 0.578  0.167  1.000 |
| Brain MRI | n=24 | n=9 | n=14 |  |
| Normal | 9 (37.5%) | 4 (44.4%) | 4 (28.6%) | 0.657 |
| Medial temporal lobe | 15 (62.5%) | 5 (55.6%) | 10 (71.4%) | 0.657 |
| Other | 2 (8.3%) | 0 | 2 (14.3%) | 0.502 |
| PET/CT | n=8 | n=1 | n=7 |  |
| Abnormal | 6 (75%) | 0 | 6 (85.7%) | 0.250 |

GAD: glutamic acid decarboxylase

**Table 2.** Treatments and outcome in anti-GAD encephalitis.

GAD: glutamic acid decarboxylase; IVIG: Intravenous Immunoglobulin; ASM: anti-seizure medicine

|  | Group 1  (n=11) | Group 2  (n=19) | P |
| --- | --- | --- | --- |
| Early immunomodulatory therapy (<6m) | 9 (81.8%) | 8 (42.1%) | 0.057 |
| IVIG | 8 (72.7%) | 16 (84.2%) | 0.641 |
| Start time (/m) (media, range) | 4.25 (1-24) | 7.5 (0.5-144) | 0.291 |
| Steroid | 10 (90.9%) | 16 (84.2%) | 1.000 |
| Start time (/m) (media, range) | 4.25 (1-60) | 8 (0.5-288) | 0.517 |
| Steroid duration (/m) (media, range) | 4.0 (0.3-10.7) | 4.4(0.7-13.6) | 0.722 |
| Immunosuppressants duration  (/m) (media, range) | 15 (6-36) | 21 (2-34) | 0.563 |
| GAD Ab titer  Decreased  Unchanged/increased  Decreased-->Increased | n=9  4 (44.4%)  2 (22.2%)  3 (33.3%) | n=16  7 (43.7%)  7 (43.7%)  2 (12.5%) | 0.600 |
| ASM  Number of ASM (median, range) | 9 (81.8%)  1 (1-3) | 17 (89.5%)  2 (1-4) | 0.358  0.040 |
